# Knowing the reproductive biology and reproductive success of *Scrophularia oxyrhyncha* Coincy as a first step for its conservation

**DOI:** 10.1186/s40529-025-00467-x

**Published:** 2025-07-02

**Authors:** Tomás Rodríguez-Riaño, Eusebio López, Josefa López, José Luis Pérez-Bote, Belén Núñez, Francisco Javier Valtueña, Ana Ortega-Olivencia

**Affiliations:** 1 Departamento de Biología Vegetal, Ecología y Ciencias de la Tierra, Área de Botánica, Facultad de Ciencias, Universidad de Extremadura, Badajoz, Spain; 2 Departamento de Anatomía, Biología Celular y Zoología, Área de Zoología, Facultad de Ciencias, Universidad de Extremadura, Badajoz, Spain; 3 Centro de Investigaciones Científicas y Tecnológicas de Extremadura, Área de Enología, Instituto Tecnológico Agroalimentario de Extremadura, Badajoz, Spain

**Keywords:** Cattle grazing, Endemic species conservation, Flowering/pollinator mismatch, Geitonogamy, Phenological disturbance

## Abstract

**Background:**

*Scrophularia oxyrhyncha* (Scrophulariaceae), endemic to southwest Spain, is one of the species of the genus whose distribution across the Iberian Peninsula is most limited. In this study, we analyzed its reproductive system by evaluating its fruit and seed set after different treatments, floral phenology, reproductive success, and the predation level of two populations in the Extremadura region (Spain), inhabiting different substrates (quartzitic in the San Serván population, granitic in the Cornalvo population) between 2019 and 2022.

**Results:**

*Scrophularia oxyrhyncha* presents protogyny, it is self-compatible but requires the presence of pollinators to produce offspring which, in quite a high percentage, would result from geitonogamous pollinations. The two populations did not differ significantly in their reproductive behaviour throughout the years studied, and both presented a spring synchronous pattern, somewhat longer in the San Serván population; this was probably due to the existence of two short second flowering periods caused by sheep predation suffered by individuals. The main threat to the populations was cattle grazing and, to a lesser extent, caterpillars.

**Conclusions:**

Cattle grazing decreased reproductive success and altered the floral display in such a way that there was a flowering time-pollinator activity mismatch. The decline in reproductive success due to vertebrate predation depended on the time at which such predation occurred, with the most severe being late predation, that is, after peak flowering. Finally, we recommend that the pertinent authorities adopt measures to ensure the conservation and survival of the populations of this endemic species in Extremadura.

**Supplementary Information:**

The online version contains supplementary material available at 10.1186/s40529-025-00467-x.

## Background

The reproductive system varies enormously in flowering plants, from those that do not require the presence of pollinators (obligate selfing) to systems in which pollination vectors are essential (obligate cross-fertilization), with several modes in between, sometimes known as mixed mating systems (Goodwillie et al. 2005). The reproductive system is one of the key biological factors affecting genetic diversity, abundance, and distribution patterns of populations in plants (Demauro 1993; Castro et al. 2008; He et al. 2024). In obligate cross-fertilization and mixed breeding systems, size of population usually plays a key role, because animal-mediated pollination, along with seed dispersal, are the most important processes (Calviño-Cancela et al. 2012; Neuschulz et al. 2016; Teixido et al. 2022). Therefore, they are essential for evolution, diversification, reproduction and plant maintenance (Briggs 2009; Pierce et al. 2018; Chomicki et al. 2019; Wei et al. 2021). Considering endangered or endemic species and/or those with a narrow range, these systems are crucial since they can be the cause of this status when one of the two processes (pollination or dispersal) fails (Swarts and Dixon 2009).

Knowledge of the reproductive system in endemic plants is even more relevant in areas with major anthropic disturbances (fires, crops, habitat fragmentation, grazing disturbance, introduction of alien and/or invasive plants, among others) since it has been demonstrated that they negatively affect mostly plant-pollinator interactions (Eckert et al. 2010; Chen et al. 2022; Teixido et al. 2022). Thus, this type of study is essential as a first step to deliver both ex situ and in situ conservation plans for endangered species (Astuti et al. 2018; Tang et al. 2020) and to maintain plant biodiversity and ecosystem functioning (Neuschulz et al. 2016).

Shifts in flowering phenology have the potential to alter ecological interactions, to the detriment of one or more interacting species. Recent models predict that a detrimental phenological mismatch may increasingly occur between plants and their pollinators. This flowering time-pollinator activity disruption provokes pollen limitation, i.e., flowers receive insufficient pollen grains thus reducing plant reproductive success, which could be due to a range of ecological perturbations, such as climate change, habitat fragmentation, etc. (Knight et al. 2018), and livestock grazing (Vázquez and Simberloff 2004); this phenomenon is expanding quickly and could endanger many species, especially those endemic or rare species with limited distribution and highly fragmented populations (Simon et al. 2001; Domínguez Lozano et al. 2013).

That flowering time-pollinator activity mismatch could reduce plant reproductive success by decreasing the pollination rate. Such disruption is considered one of the consequences of climate change (Wall et al. 2003; Memmott et al. 2007; Hegland et al. 2009), but it may also be due to other factors, such as anthropogenic degradation of vegetation by livestock grazing that also causes a decrease in reproductive success through the consumption of vegetative and reproductive structures. This degradation affects plant species density, population density and dynamic, and alters the floral display in such a way that there is a mismatch between flowering and pollinator activity (Aschero and Vázquez 2009; Zhang et al. 2017; Chen et al. 2022).

This paper focused on *Scrophularia oxyrhyncha* Coincy (Scrophulariaceae), most of whose populations live on quartzitic substrate as opposed to other less abundant populations that inhabit on granitic substrate. This species is endemic to southwest Spain (in Sierra Morena and south of the Montes de Toledo; Ortega-Olivencia 2009) and is considered “vulnerable” in the regions of Extremadura (DOE 2001; Vázquez et al. 2010), Andalusia (Cabezudo et al. 2005) and Castilla-La Mancha (DOCM 2001). At national level, the Red List of Spanish Vascular Flora categorizes it as “data-deficient” taxon (Moreno 2008, 2011). According to the IUCN, a taxon is “Vulnerable (VU)” when the best available evidence indicates that it meets any of criteria A to E, and is, therefore, considered to be at high risk of extinction in the wild (IUCN 2012). In the case of the Extremadura region, that category is applied to those species at risk of downgrading to a more alarming status (“sensitive to the alteration of their habitat” and “in danger of extinction”) in the immediate future if the adverse factors acting on them are not corrected. It is also indicated that cataloging this species requires the drafting of a Conservation Plan and, where appropriate, the protection of its habitat. These actions are yet to be carried out in Extremadura, except that one of the populations is located in a Natural Park (Cornalvo). The main threats to this species are grazing, desiccation and loss of the adjacent forest mass, suggesting that conservation measures should include the isolation of some populations from logging or livestock exploitation, and the establishment of monitoring models (Cabezudo et al. 2005; Vázquez et al. 2010).

The main goal of this work was to study the reproductive biology of this species to know its reproductive peculiarities and to discern a potential threat that could cause deterioration or decline in the existing populations and, in this way, to provide data for the competent authorities to inform the actions required to conserve this endemism. An additional aim was to study its flowering phenology, as a process closely related to pollination and, obviously, to sexual reproduction. The study was developed between 2019 and 2022 in two populations in the Badajoz province (Extremadura, in southwest Spain).

## Materials and methods

### Species studied

*Scrophularia oxyrhyncha* is a species endemic to southwest Spain (Sierra Morena and south of the Montes de Toledo) with most of its populations concentrated in the province of Badajoz (Extremadura), and a few in neighboring provinces (Córdoba and Ciudad Real, in the Andalusia and Castilla-La Mancha regions, respectively) (Ortega-Olivencia and Devesa 1993a; Ortega-Olivencia 2009). It is a plant with low capacity for interspecific competition so, in general, all known populations have a reduced number of mature individuals growing occasionally in isolation or closely grouped in areas with little or no competition from other species, hence their location, generally in crevices, cavities or craggy places (Ortega-Olivencia 2009; and authors’ personal observations). In addition, it should be noted that it is one of the endemic species of the genus whose distribution across the Iberian Peninsula and Mediterranean region is among the most limited (Ortega-Olivencia and Devesa 1993a).

The two populations studied do not have the same ecology, and show some differences in habit type, among other features. One population inhabits quartzitic substrates, and their individuals have greater longevity than the other, which occupies granitic substrates.

**Fig. 1:**
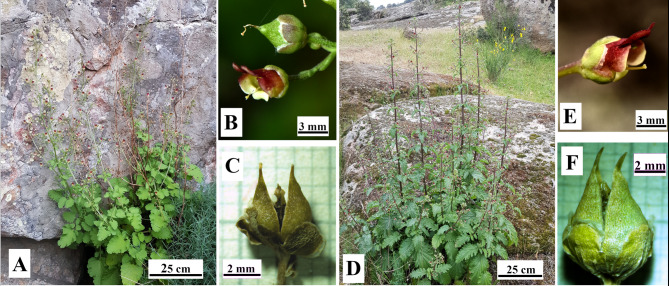
*Scrophularia oxyrhyncha*. **A**-**C** Sierra de San Serván (quartzite substrate), **D**-**F**, Cornalvo Natural Park (granite substrate). **A**, **D** general aspect of a flowering individual. **B**, **E** detail of flower. **C**, **F** detail of fruit

The individuals from the quartzitic population are glabrous or glabrescent, perennial, rhizomatous herbs up to 130 cm tall with lustrous leaves, mostly undivided, or pinnatisect in the lower ones, and endowed with a large, broadly ovate or suborbicular terminal segment and 1–2 smaller basal segments. The flowers are arranged in mostly alternate whorls of compound and pedunculated dichasial cymes of up to 16 flowers forming more or less erect inflorescences (Fig. 1A). The corolla is bilabiate, intermediate in size in the genus (4.9–7.5 mm), yellowish-green in color, with a purplish-reddish upper lip (Fig. 1B). The androecium comprises four fertile stamens and a fifth sterile one, the staminode, which is suborbicular or obovate, greenish or greenish-purple. The bicarpellate gynoecium consists of a long style and a slightly bilobulate stigma. The fruit is a subconical capsule, 6–8 mm long (Fig. 1B, C) (Ortega-Olivencia 2009), which houses an average of 93.9 small (0.5–1.2 × 0.3–0.6 mm) blackish-brown seeds (Ortega-Olivencia and Devesa 1993b). The individuals from the granitic population are annual or biannual herbs up to 214 cm tall, less branched than the previous one, with longer inflorescences endowed with dichasial cymes that carry up to 30 flowers. Flowers and fruits are generally larger in size than those present in the quartzitic population (Fig. 1D-F).

The only known study on the floral and reproductive biology of this species was carried out under experimental garden conditions (Ortega-Olivencia and Devesa 1993b, c). Its flowers, which are nectariferous, possess protogyny, as in most species of the genus (Valtueña et al. 2013), and even in many genera of the old Scrophulariaceae (e.g., *Veronica*, *Pedicularis*; Kampny 1995), the male phase lasting longer (2.30 days) than the female (1.10 days). The fruit and seed set after natural pollination was about 71% and 68%, respectively, and null after inflorescence isolation to the pollinators, highlighting the importance of pollinators in the sexual reproduction of this species. However, no experiments were performed to determine whether it was self-incompatible. No studies were carried out on diaspores dispersal, although presumably seeds will be expelled from capsules when fruiting branches are shaken by the wind and/or animals (semachory), as in *Scrophularia canina* L. (Rodríguez-Riaño et al. 2017, 2019). Flowering occurs in winter-spring (February to May); fructification takes place approximately one month after flowering, and seed germination in autumn (Ortega-Olivencia and Devesa 1993a; Ortega-Olivencia 2009).

### Populations studied

**Fig. 2:**
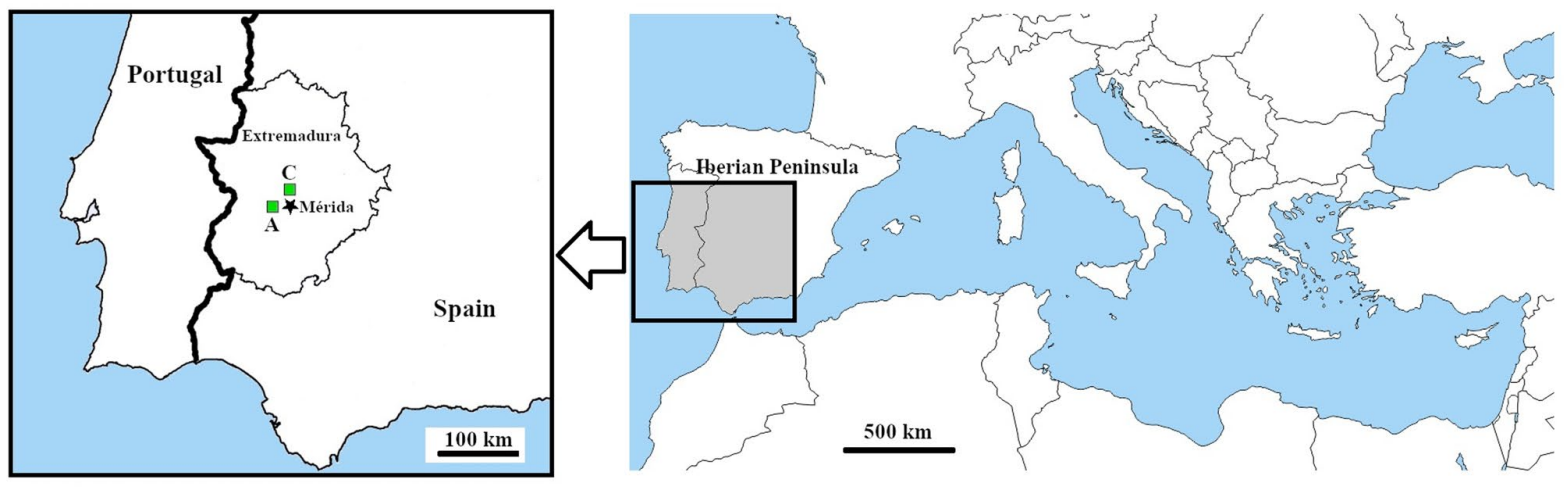
Location of the two populations studied of *Scrophularia oxyrhyncha* (A Sierra de San Serván, C Cornalvo Natural Park)

Two populations located in the southwest of the Iberian Peninsula (Badajoz, Extremadura, Spain) were studied (Fig. 2). The first population is situated in the Sierra de San Serván (henceforth, San Serván), near the city of Merida and grows on a quartzite substrate; the second population, located in Cornalvo Natural Park (henceforth, Cornalvo), develops on a granite substrate; they are about 30 km apart.

The San Serván population (Fig. 2) is characterized by the presence of typical individuals of the species (see above), occupying an area of approximately 12 ha. Individuals grow at the base or between cracks of a quartzite crag located at the crest of the mountain range on somewhat nitrified soils, above 410 m asl (38°51’59.0"N-6°26’30.8"W). The Cornalvo population (Fig. 2) forms part of the Network of Protected Natural Areas of Extremadura and the Natura 2000 Network. By contrast, this population is characterized by the presence of annual or biannual individuals occupying an area of approximately 0.7 ha. Individuals grow at the base or between cracks of a rocky granite outcrop on sandy and somewhat nitrified soils, above 310 m asl (39°01’50.0"N-6°10’59.4"W). Both populations are situated on the Mesomediterranean floor and are exposed to grazing by livestock or wild animals, or to human disturbance.

The zonal vegetation of San Serván is constituted by a Mediterranean scrubland rich in species, which is replacing a holm oak forest (*Quercus rotundifolia* Lam.) with the occasional presence of cork oak (*Quercus suber* L.), mainly as a result of a forest fire that swept the area in summer 2017. This area is frequently grazed on by sheep and/or goats. The zonal vegetation of Cornalvo is constituted by a meadow with holm oaks and brooms, with vegetation that is more open than in San Serván. Most of the *S. oxyrhyncha* individuals grow at the base, between cracks or nested cavities of the rocky outcrop, and coexist with herbaceous species, as in the San Serván population. This area is frequently grazed on by cows.

Taking as reference the Meteorological Station of Mérida (intermediate distance from both studied populations; see Fig. 2) and considering the years of study (2019–2022) plus the previous year, the annual mean temperature was approximately 17–18ºC, with temperatures ranging from (-5.0) 2.0ºC in January-February to 37.7(44.8ºC) in August. The total annual precipitation was approximately 442.9 mm. The maximum precipitation occurred in March 2018 (140.3 mm) and the minimum in the summer months (June-September), with some months without any precipitation. Approximately 40% and 35% of precipitation occurred during March-May (monthly average about 53 mm) and during September-November (monthly average about 50 mm), respectively (AEMET 2024). However, the population of San Serván endures a colder atmosphere due to location in a mountainous area, while the Cornalvo population lies on a more or less flat area, though close to a stream (arroyo de las Muelas).

### Reproductive system

Fieldwork was carried out during the flowering (February-July) and fruiting (June-July) seasons of 2019 and 2021. To evaluate the reproductive system of *S. oxyrhyncha*, we carried out (1–2 times a week) several pollination tests in two different groups of individuals (Gr. 1 and Gr. 2) in each of the populations.

*Gr. 1*, in which four types of tests were performed: spontaneous self-pollination (SSP), hand-geitonogamous pollination (HGP), hand-cross pollination (HCP) and agamospermy (AG). These four treatments were compared with an open-pollination test (control, C).

*Gr. 2*, in which a forced cross pollination treatment (FCP) was performed and compared with a forced control open pollination (CFCP). In this case, and for the Cornalvo population, the considerable size of the inflorescences led us to select small individuals in order to carry out the experiments properly.

We selected and marked 15 different individuals per group of treatment (Gr. 1 and Gr. 2) in each population and in each year of study. For Gr. 1 plants, each individual selected was divided into 5 inflorescence groups, one for each treatment (including control) and, for Gr. 2, each chosen individual was divided into 2 inflorescence lots. Flower marking consisted of painting the calyx/pedicel with a different color plastic paint for each treatment.

Every time the population was visited, all the inflorescences with available flowers were selected in order to perform the following treatments.

#### Spontaneous self-pollination (SSP)

All pre-anthesic flowers were marked and not manipulated, then each inflorescence was isolated from pollinators by placing them in white nylon bags and retained until the last flower wilted.

#### Hand-geitonogamous pollination (HGP)

15–20 flowers in the female phase (i.e., newly opened flowers) per inflorescence group were hand-pollinated with sufficient pollen deposited on the stigma from other flowers of the same individual; the inflorescences were then bagged as in the previous treatment.

#### Hand-cross pollination (HCP)

As in the previous treatment, but pollination was performed with pollen from flowers of different individuals located at least 10 m from the recipient plant.

#### Agamospermy (AG)

The stigmas of 15–20 flowers were excised and bagged, as in the previous treatments.

#### Open or control pollination (C)

Flowers were subjected to natural pollination, that is, there was no manipulation whatsoever.

#### Forced cross-pollination (FCP)

15–20 flowers in the female phase were selected, emasculated and marked, and the rest of flowers in the male phase of each plant (potentially pollinated were considered as control for forced cross-pollination, CFCP) were emasculated to avoid geitonogamous pollination of the flowers in the female phase; then each individual was kept for open-pollination (i.e., not bagged).

In relation to the C and CFCP treatments, we used two different methodologies. In 2019, all flowers from the control inflorescences were considered. By contrast, in 2021, each time a manual pollination experiment was carried out, a similar number of flowers was marked on the control inflorescence. In 2019, considering the complete inflorescence, the number of fruits obtained was very high, which meant a lot of time for seed counting. To avoid this, in the 2021 controls, each time manual pollination was performed on their respective individuals, a similar number of flowers were selected in the control treatment. This different methodology did not significantly influence the results obtained in both types of controls between seasons [San Serván 2019 vs. 2021 (Control: F = 0.237, df = 1, *P* = 0.630; CFCP: F = 2.290, df = 1, *P* = 0.142); Cornalvo 2019 vs. 2021 (Control: F = 2.551, df = 1, *P* = 0.124; CFCP: F = 1.204, df = 1, *P* = 0.284)].

The fruit set (ratio between the number of mature fruits with at least one viable seed and the number of flowers selected; Fr/Fl ratio) and seed set (ratio between the number of viable seeds and the number of ovules per fruit; S/O ratio) were both determined. Ripened fruits were collected by cutting them before dehiscence, conserved in opaque envelopes and transported to the laboratory. Viable (well-formed) seeds per fruit were counted under a stereomicroscope. In 2019, the control seed set was calculated, considering only 10 mature fruits of all those collected that were distributed evenly along the fruiting branch. Because ovules could not be counted in mature fruits, the number of ovules per ovary to calculate S/O ratio was accounted for by 10 randomly selected flower buds of each experimental individual under a light microscope.

### Flowering phenology

During the 2019–2022 seasons, 30–40 individuals in the San Serván and Cornalvo populations were selected and tagged for a weekly count of the number of open flowers in both phases (male and female). With these data we estimated the geitonogamy probability per population and season, as the difference between the percentages of flowers in the male and female phases (henceforth, D); values of D equal to 0 corresponding to a total geitonogamy, and 100 to its absence, according to Navarro-Pérez et al. (2019). In addition, we also determined the degree of flowering synchrony by using the Augspurger coefficient (1981, 1983) adapted to weeks (sampling frequency in this study). Synchrony for an individual *i* is *X*_*i*_ = [1/(*n*-1)](1/*f*_*i*_)Σ*e*_*j≠i*_, where *j≠i* is the number of weeks in which individuals *j* and *i* coincide in flowering; *fi* is the number of weeks that individual *i* is in flowering, and *n* is the number of individuals studied per population. The population synchrony (Augspurger 1981, 1983) was calculated as *Z* = (1/*n*) Σ*X*_*i*_. Both the *Xi* and *Z* values range from 0 to 1, with 1 representing maximum synchrony.

### Plant predation and reproductive success

To ascertain the degree of plant predation by different agents (herbivorous vertebrates and invertebrates), we made observations and noted the type of damage caused to each individual plant. In the case of vertebrates, we differentiated three predation types: (a) early predation, the individual suffered predation before reaching peak blooming; (b) late predation, occurring after peak blooming, and (c) double predation, the individual suffered both early and late predation.

In the case of predation by invertebrates, levels were defined based on the degree of damage to reproductive plant parts, following Rodríguez-Riaño et al. (2022): (a) little or no damage to reproductive units (flowers and fruits); (b) low predation, less than 25% of reproductive units predated; (c) high predation, between 25 and 75% of reproductive units predated and (d) severe damage, more than 75% of reproductive units predated.

In terms of individual reproductive success, we considered it as fruit harvest (i.e., the total number of fruits produced per individual at the end of the season). Each May during the 2019–2022 seasons, we counted the inflorescence number in all the individuals selected for phenology analysis. Additionally, the number of floral cymes in 30–40 inflorescences in both populations was recorded. At the end of the fruiting season, the fruits produced in 10 randomly selected cymes per individual were collected. Finally, the total fruit production per individual, after multiplying the average number of fruits per cyme and the number of inflorescences per individual, was calculated. The total number of fruits per individual in the San Serván population, if the individual had less than three inflorescences, was counted.

During 2020 due to COVID-19 confinement, it was impossible to carry out any of the studies developed in this work (reproductive system, reproductive success, plant predation, etc.), since the populations could not be visited for almost two and a half months. The scarce data obtained are presented but were not considered for analysis.

### Statistical analysis

All analyses were performed using the statistical package SPSS version 27.0.1. Normality of variables was checked using the non-parametric Kolmogorov-Smirnov test with Lilliefors correction, and homoscedasticity was assessed using the Levene test.

The fruit and seed sets were analyzed using a generalized linear model (GLM) fitting to a normal distribution with a logarithmic link function. In both cases, the fruit and seed set, treatment and year were used as principal factors.

Before the global analysis of all treatments was carried out, we analyzed whether the methodology followed for both types of open pollination, control (C) and forced control (CFCP) influenced the outcome by applying a one-way ANOVA. As both types of open pollination were not statistically different [San Serván (fruit set: F = 0.005, df = 1, *P* = 0.944; seed set: F = 0.283, df = 1, *P* = 0.597); Cornalvo (fruit set: F = 1.294, df = 1, *P* = 0.244; seed set: F = 0.122, df = 1, *P* = 0.728)] we did not consider the forced control open pollination treatment for analysis. In the same way, the agamospermy treatment was not considered because the fruit set was null in all the individuals studied. In the SSP treatment, we used all the data obtained, although some individuals with relatively high fruiting values could be considered artifacts possibly due to the rubbing of the bag against the flowers. Neither was the seed set for SSP treatment considered for statistical analysis because only 4–5 individuals fruited with very low levels.

## Results

### Reproductive system

**Fig. 3:**
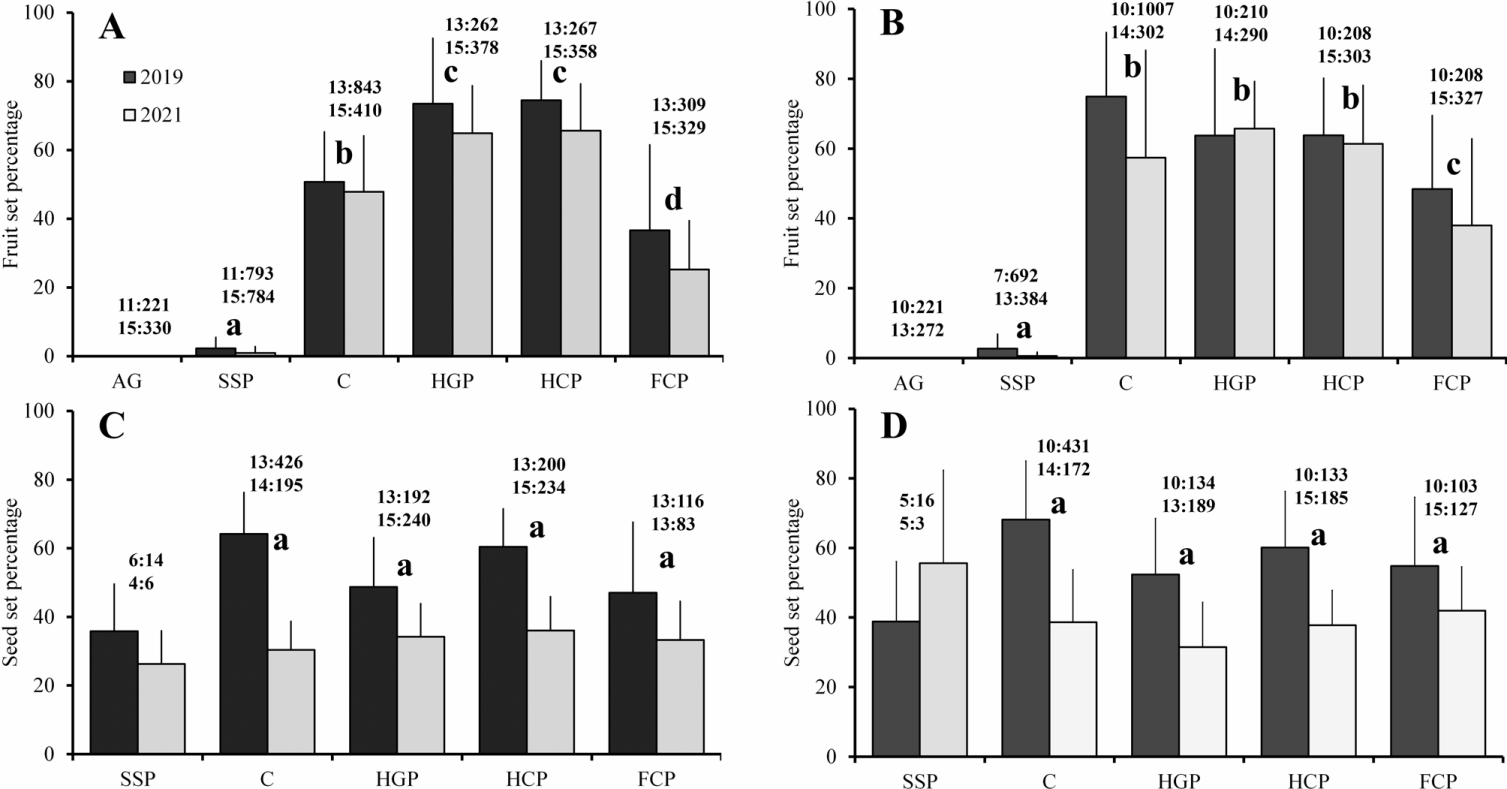
Percentage of fruit and seed sets (mean ± s.d.) in San Serván (**A**, **C**) and Cornalvo populations (**B**, **D**) of *Scrophularia oxyrhyncha.* The percentage were calculated after natural pollination (C) and five different pollination treatments (AG agamospermy, FCP forced cross pollination, HCP hand-cross pollination, HGP hand-geitonogamous pollination, SSP spontaneous self-pollination) during two seasons (2019 and 2021). San Serván, Sierra de San Serván population; Cornalvo, Cornalvo Natural Park population. Pollination treatments with different letters indicate a significant difference at the 0.001 level. Above each pair of bars is indicated the sample size (top data refers to season 2019 and bottom data to 2021): before the colon, the number of individuals considered for the statistical analyses (N) and after the colon, the number of flowers (fruit set graphs) or fruits (seed set graphs) used to calculate the mean value for each individual

The fruit set varied significantly in the treatments in both populations, remaining unaffected by either the year or the treatment*year interaction (Table 1). Spontaneous self-pollination never exceeded 12% in any of the populations or years studied. The treatments were combined in 5 groups in the San Serván population (Fig. 3A): (i) agamospermy (AG), which did not produce any fruit; (ii) spontaneous self-pollination (SSP), with a practically null fruit set (mean ± SD: 1.48 ± 2.52%); (iii) forced cross-pollination (FCP), with a fruit set of 30.51 ± 20.30%; this fruit set improved significantly in comparison to that obtained after SSP, but was significantly lower than those from (iv) the open pollination or control (C), and (v) the hand-geitonogamous pollination (HGP) and the hand-cross pollination (HCP), which were the most effective treatments. After control, the fruit set reached an average of 49.17 ± 15.26%, and although significantly higher than that of FCP, it was significantly lower than HGP (68.86 ± 16.68%) and the HCP fruit set (69.73 ± 13.21%), the last two with no significant difference between them. In the Cornalvo population (Fig. 3B) the treatment grouping (SSP, 1.34 ± 2.70; FCP, 42.15 ± 23.54%; C, 64.69 ± 27.27%; HGP, 64.88 ± 18.60%; and HCP, 62.37 ± 16.29%) was similar to those in San Serván, but with a single difference, the control did not differ significantly from the hand-pollination treatments (HGP y HCP). As year*treatment interaction was not significant (Table 1), the reproductive strategy of both populations was practically the same for the different years (San Serván: AG < SSP < FCP < C < HGP = HCP; Cornalvo: AG < SSP < FCP < C = HGP = HCP).

The seed set only varied significantly in the years for both populations, being unaffected by either the treatment or the treatment*year interaction (Table 1). In both populations, the seed set from 2019 was significantly higher than that of 2021 (Fig. 3; Table 1). Results from SSP could not be considered because only a few flowers fruited and there were not enough data available for comparison with the other treatments. In addition, these results are possibly misleading, since the production of these fruits was probably due to the rubbing of the reproductive organs of the flowers against the bags used for bagging (see statistical analysis subsection). As expected, the seed set after SSP, except for the Cornalvo population data in 2021 (see previous comment), was lower than for rest of the treatments (Fig. 3C, D).

**Table 1:** Generalized linear model (GLM) fitted to a normal distribution with a logarithmic link function to analyze the effect of treatment and year on fruit and seed set for the two *Scrophularia oxyrhyncha* populations

Variable	Factor	d.f.	χ^2^Wald	*P*
**Fruit-set**				
**San Serván**	Treatment (Treat)	4	100.365	**0.000**
	Year	1	0.126	0.723
	Year*Treat	4	2.270	0.686
**Cornalvo**	Treatment (Treat)	4	18.829	**0.000**
	Year	1	0.049	0.825
	Year*Treat	4	3.929	0.416
**Seed-set**				
**San Serván**	Treatment (Treat)	3	5.400	0.145
	Year	1	73.296	**0.000**
	Year*Treat	3	7.601	0.055
**Cornalvo**	Treatment (Treat)	3	6.020	0.111
	Year	1	48.521	**0.000**
	Year*Treat	3	3.892	0.273

Treatment: spontaneous self-pollination; hand-geitonogamous pollination; hand-cross pollination; open pollination and forced cross-pollination. For seed set, spontaneous self-pollination was not considered. Population: Sierra de San Serván (quartzite substrate) and Cornalvo Natural Park (granite substrate).Year: 2019 and 2021

Although no significant differences were found in the treatments (Table 1), it was observed that the seed set was, for both seasons and populations, lower in the HGP and FCP treatments than in those of C and HCP (Fig. 3C, D), that is, the treatment behavior, as in the fruit set, was not affected by the year. In both the fruit and seed sets, it was observed that the 2019 season was more fruitful than the 2021 season.

**Fig. 4:**
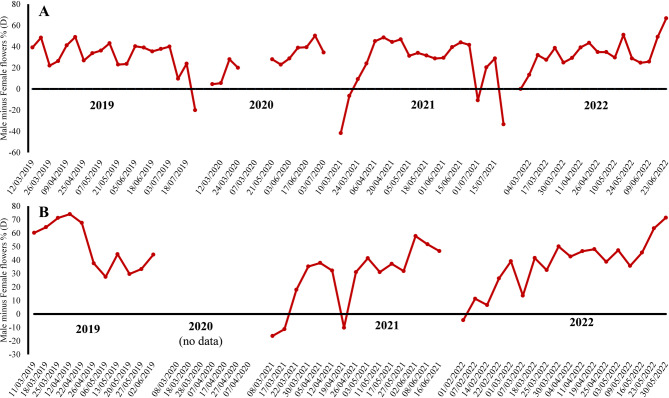
Geitonogamy probability (D) in two populations (**A** Sierra de San Serván, **B** Cornalvo Natural Park) of *Scrophularia oxyrhyncha*. For each season studied, the geitonogamy probability was measured as the difference between the percentage of flowers in the male phase and flowers in the female phase. Value of 0 corresponding to total geitonogamy and 100 to its absence

These results show that a high percentage of the offspring would have come from geitonogamous pollinations, which is corroborated by the high/median probability of the existence of geitonogamy (about 50%) in both populations during most of the phenological period (many more male flowers than female ones), although that probability was higher at the beginning and/or end of the phenological period (Fig. 4). The likelihood of geitonogamy was usually higher throughout the entire flowering period in San Serván (values almost always closer to 0) than in Cornalvo, which was consistent with the structure of the individuals belonging to San Serván (perennial herbaceous with many small inflorescences per individual) compared to Cornalvo (annual or biennial herbaceous with few large inflorescences per individual).

### Flowering phenology, reproductive success and plant predation

**Fig. 5:**
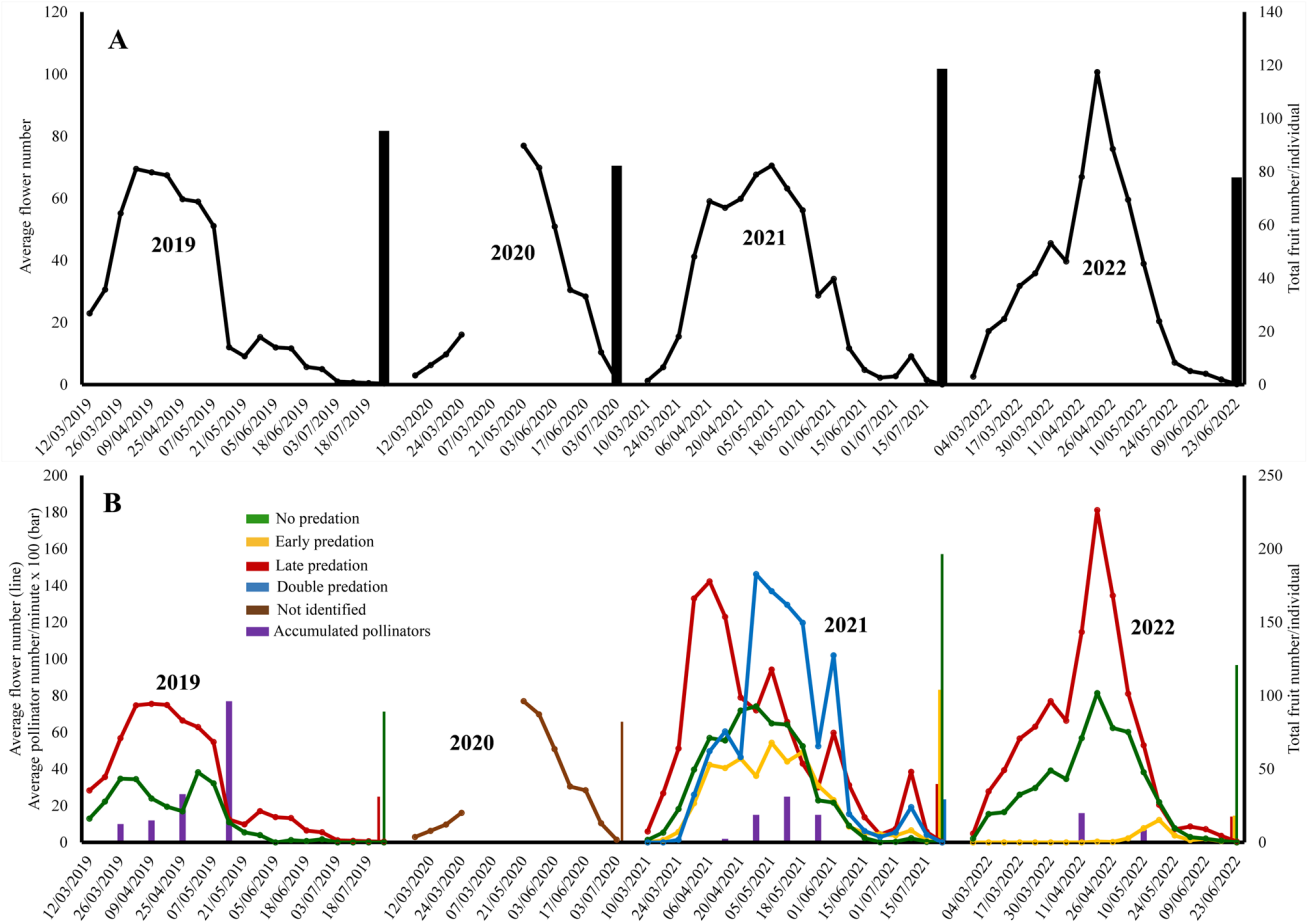
Average of flowering phenology (lines), fruit harvest (non-purple solid vertical lines) and average number of pollinators per minute of censuses (purple vertical lines) of *Scrophularia oxyrhyncha* in Sierra de San Serván population. **A** considering all selected plant individuals, **B** considering groups of plant individuals after different vertebrate predation type. Seasons of study: 2019–2022

**Fig. 6:**
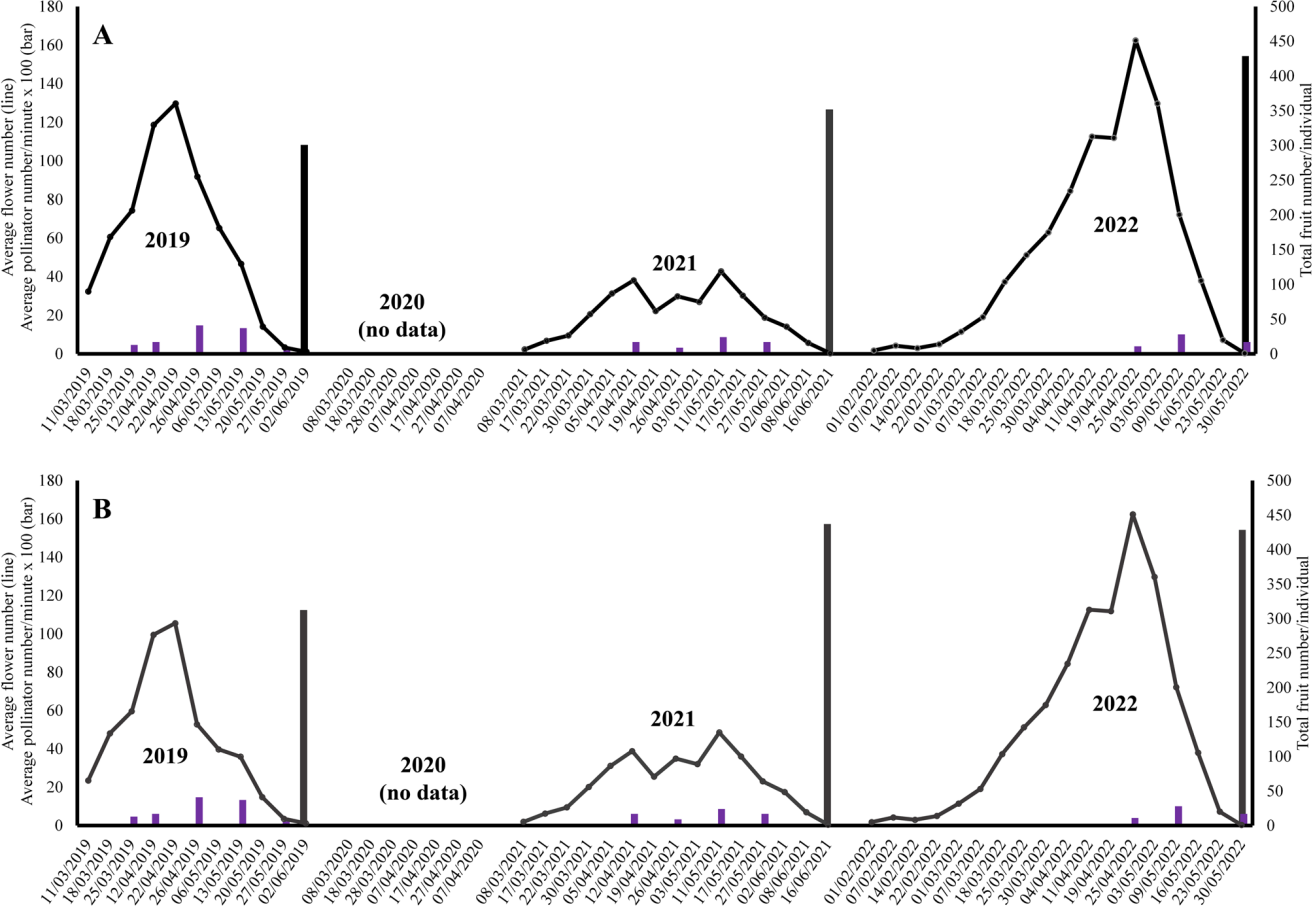
Average flowering phenology (lines), fruit harvest (black vertical lines) and average number of pollinators per minute of censuses (purple vertical lines) of *Scrophularia oxyrhyncha* in Cornalvo Natural Park population. **A** considering all selected plant individuals, **B** considering all plant individuals except those totally predated by invertebrates (caterpillars). Seasons of study: 2019–2022

Both populations had a spring flowering pattern (Figs. 5 and 6). In the San Serván population it lasted for almost 5 months (Fig. 5A), and almost four months in Cornalvo (Fig. 6A). In San Serván, the phenological pattern showed three flowering peaks (Fig. 5A): a big first main peak and two much smaller ones, the latter at the end of the phenological period, which was clearly visible in the 2019 and 2021 seasons. By contrast, in Cornalvo there was no late flowering peak (Fig. 5). Flowering phenology was highly modified by sheep predation (see Fig. 5B), but not so much by caterpillar predation (Fig. 6B vs. Fig. 6A).

Regarding flowering synchrony, individuals in both populations showed similar synchronic phenological patterns (see Table S1). This synchrony was especially affected by early predation (see season 2022 in Table S2, although these data must be treated with caution due to the low number of individuals analyzed).

**Fig. 7:**
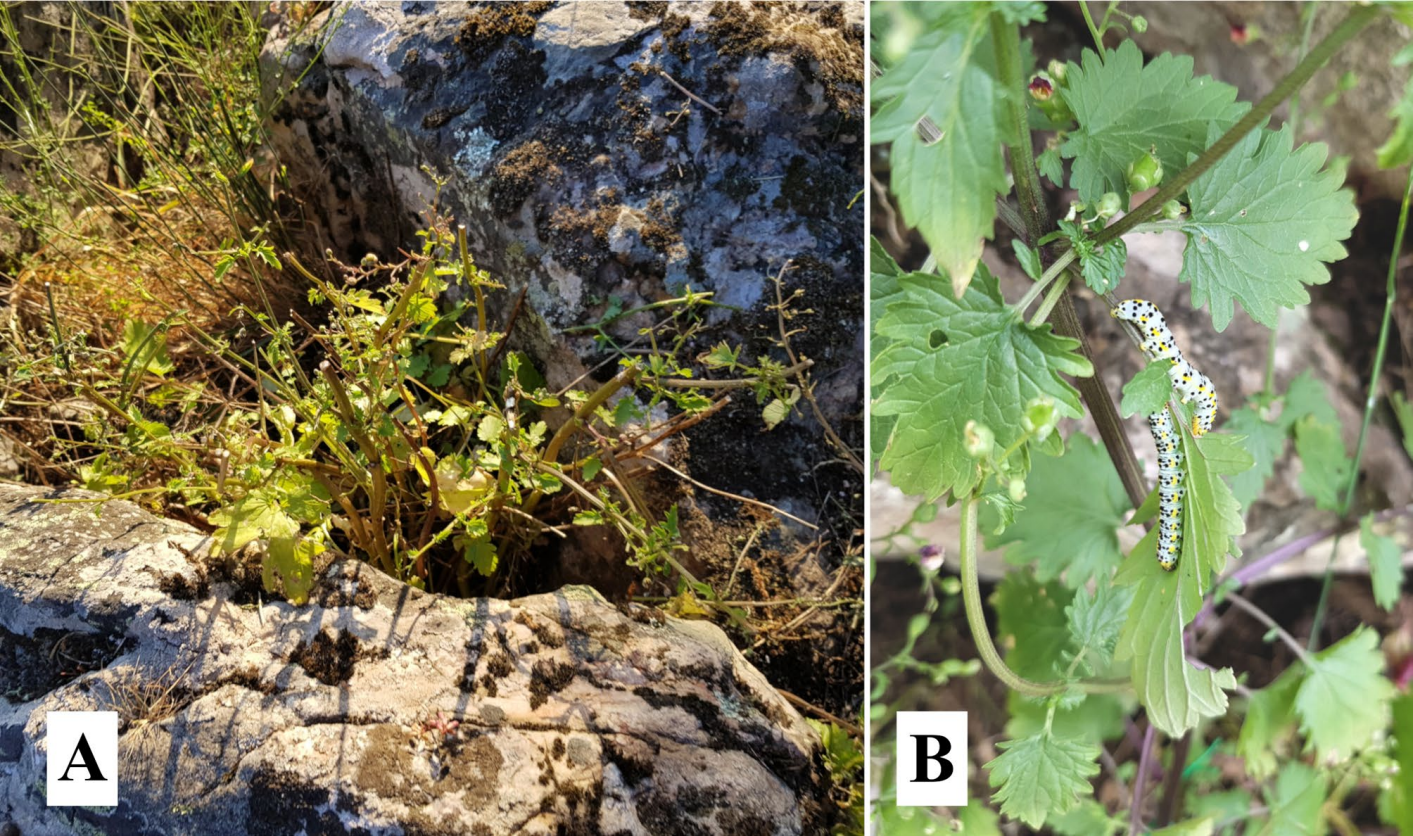
*Scrophularia oxyrhyncha* predation. **A** individual predated by sheep (Sierra de San Serván population), **B** individual with two caterpillars feeding on leaves (Cornalvo Natural Park population)

Both populations differed in relation to predation type. In San Serván, cattle grazing, specifically by sheep, predominated (Fig. 7A) vs. caterpillars (almost null). On the contrary, in Cornalvo herbivory by invertebrates [caterpillars, *Sargacucullia scrophulariae* (Denis & Schiffermüller) 1775)] was the most prominent (Fig. 7B). The main damage to plants by sheep was due to grazing, which caused stem and inflorescence breakage; it even killed some small individual plants. Vertebrate predation had a much greater influence than the invertebrate type on both reproductive success and flowering phenology.

Plant predation in both populations differed over the years studied. Late sheep predation in San Serván in 2019 was very strong, with 34 of 39 individuals suffering some degree of predation. By contrast, during the 2021 season, practically half the individuals (17 of 37) avoided any predation. Caterpillar predation in Cornalvo, as a rule, caused mild effects on plant vegetative parts (e.g., a partial defoliation), without leading to the death of any individual. In this sense, only 2021 stood out, as when 8 individuals out of a total of 41 presented very severe damage (completely predated individuals, i.e., there was no fruit formation).

Reproductive success (i.e., mean fruit harvest per individual) in San Serván ranged between 77.87 and 118.66 fruits (Fig. 5), 2021 seeing the highest fruit harvest, while in Cornalvo it varied between 303.60 and 428.59 (Fig. 6). Predation by vertebrates reduced this success considerably more than predation by invertebrates (see Figs. 5 and 6).

Considering vertebrate predation, reproductive success was largely determined by degree and type. As expected, the greatest reproductive success was enjoyed by those individuals not subjected to any form of predation, followed by the individuals that suffered early predation (i.e., before reaching peak flowering). Lastly came those that underwent late or double predation (i.e., early and late; Fig. 5B). The late predation high point, during 2019, reduced reproductive success as predation indiscriminately eliminated almost all produced fruits (Fig. 5B). Luckily, most of these individuals sprouted again and even produced new flowers, however these did not bear fruit at that time because there were hardly any pollinators (Fig. 5B), which had abandoned the population because of insufficient floral rewards, or which visited other flowering plants with higher reward levels. Double predation (early and late) in 2021 and 2022 caused the greatest reduction in reproductive success along with late predation, between 5 and 7 times lower compared to those individuals that did not suffer any predation, while early predation alone reduced fruiting by approximately twice as much as those that suffered no predation at all (Fig. 5B).

## Discussion

*Scrophularia oxyrhyncha* is one of the endemic Iberian or Mediterranean species of the genus whose distribution is most restricted, with the majority of populations concentrated in the province of Badajoz, just a few more in Cordoba and Ciudad Real provinces (Ortega-Olivencia 2009; Ortega-Olivencia and Devesa 1993a). Therefore, this study is justified by the need to conserve the species, as is gathering information on the state of its populations. Our results show that in terms of reproduction, the species is self-compatible, and needs pollinators for sexual reproduction, presenting a mixed reproductive system. Due to its self-compatibility and the presence of numerous flowers opening at the same time in female and male states by inflorescence and individual (asynchronous protogyny), the potential level of geitonogamy throughout the flowering period ranged from intermediate to high. In addition, it has been demonstrated that predation altered (negatively) the phenology of the flowering and reproductive success and, indirectly, the level of geitonogamy.

### Reproductive system

To carry out the study, two populations found in different geological substrate types (quartzites in the San Serván population, granites in the Cornalvo population) were selected in order to observe potential differences. During their development we perceived that the individuals of the two populations varied in longevity, size, branching level and in other features, especially in terms of flower size, inflorescences and fruits. Therefore, we can deal with plants that sharing characteristics with the species type according to Coincy (1898), and which inhabits on quartzitic substrate (San Serván population) and other individuals deviating from the most relevant diagnostic characteristics of the species, and which develop on granite substrate (Cornalvo population). The latters are larger and more column-shaped than the first individuals. In both cases, the individuals mainly exist between the cracks of the granite or quartzite rocks. This could be due to the difficulty that this species has, as with other *Scrophularia* species (e.g., *Scrophularia arguta* Aiton), in competing with other plant species, being relegated to the most extreme and specialized sites. Both types of individuals present a low capacity for interspecific competition so, in general, all known populations of the species have a reduced number of mature individuals, growing occasionally in isolation or closely grouped in areas with little or no competition from other species, hence their location, generally in crevices or in craggy places (Ortega-Olivencia 2009 and authors’ personal observations).

Both types of *S. oxyrhyncha* plants present protogyny and are self-compatible but require the presence of pollinators to produce offspring (null, or almost null after SSP, in the latter case probably due to the rubbing of the bags against the flowers) just as most *Scrophularia* species studied so far (e.g., Shaw 1962; Dalgaard 1979; Ortega-Olivencia and Devesa 1993b; Navarro-Pérez et al. 2017). Therefore, self-compatibility simply reinforces this reproductive character in *Scrophularia* (only one case of self-incompatibility is known: *Scrophularia fontqueri* Ortega Oliv. & Devesa; Ortega-Olivencia and Devesa 1998), which reaches its end in other species of the genus that are clearly autogamous (e.g., the annuals *Scrophularia peregrina* L. and *S. arguta*) in which protogyny does not operate. In fact, in *S. arguta*, which has homogamous flowers (Stiefelhagen 1910; Dalgaard 1979), the fruit set after SSP treatment was no different from the control (Rodríguez-Riaño et al. 2022). On the other hand, in our species the quantity of available pollen decreased due to the emasculation of flowers in the female phase, which means that they did not provide pollen in the male phase (FCP treatment), therefore the fruit set reduced significantly compared with those treatments without this limitation (HGP, HCP), and even the control.

At the reproductive level, the two populations did not differ significantly in their behaviour throughout the years studied, with the fruit and seed set in 2019 being greater than in 2021. In both populations there was a clear probability of geitonogamy, greater in the San Serván population than in Cornalvo; this is probably due to the structure of individuals, highly branched and with many small inflorescences per individual in San Serván, and lightly branched and with few large inflorescences per individual in Cornalvo. Geitonogamy is a fairly widespread type of reproduction in plants (Faegri and van der Pijl 1979; Jones and Little 1983; Robertson 1992) which presents negative implications such as inbreeding depression and/or loss of pollen exports to other individuals in self-compatible plants (de Jong et al. 1993). Intermediate levels of geitonogamy have recently been cited in other species of *Scrophularia* with distribution in the western Mediterranean region (e.g., *Scrophularia grandiflora* DC. and *Scrophularia sambucifolia* L.), and in which the percentage of geitonogamy was reduced by increasing the age of inflorescences (Navarro-Pérez et al. 2019). In general, predation usually has very negative effects on the population dynamics of many species (Aschero and Vázquez 2009; Chen et al. 2022; Rodríguez-Riaño et al. 2022), and in some cases it can even lead to the disappearance of populations. However, so far there are few demonstrated cases that reflect its influence on the level of geitonogamy (e.g., Strauss et al. 1996, 1999; Elle and Hare 2002; Liao et al. 2013). In relation to this level of geitonogamy, herbivory could reduce it by decreasing the number of flowers available to be successively visited by a pollinator within the same plant (de Jong et al. 1993; Liao et al. 2013).

### Flowering phenology, reproductive success and plant predation

Regarding flowering, both populations presented a spring synchronous pattern, somewhat longer in the San Serván population, probably due to the two short second flowering periods caused by sheep predation suffered by individuals. These second flowering periods were totally fruitless, presumably as a result of fewer pollinators due to the flowering/pollinator mismatch. Furthermore, the longer flowering period in San Serván could be sustained because *Scrophularia* individuals are more protected by the surrounding vegetation (higher plant species density) and by the “mountain” effect that would imply a cooler environment and an increase in inflorescence longevity.

In both populations the presence of pollinators is crucial for maintaining the genetic variability through gene flow via pollen, but there also needs to be an absence of disruption between the flowering time of a plant species and pollinator activity. Such disruption can considerably reduce the reproductive success of individuals, which could undermine the survival of these populations (Wall et al. 2003; Memmott et al. 2007; Aschero and Vázquez 2009; Hegland et al. 2009; Zhang et al. 2017; Chen et al. 2022). This negative impact on species is intensified in endemic species, and even more so in those consisting of a few small and isolated populations. The pollinator-flowering time disruption was first observed in the San Serván population, where the predation of a large number of individuals before and after the flowering peak (early and late predation, respectively) caused a considerable decrease in the reproductive success of these preyed-upon individuals, which was greatly reduced or even completely null, as in other species (see Simon et al. 2001; López-Sánchez et al. 2016; Domínguez Lozano et al. 2020). Intense predation by sheep observed in certain individuals, during flowering or even the fruiting period, means that, by not having to expend resources on the maintenance of the fruits and seeds (null or almost null fruit set after predation), they then sprouted and began a new late flowering period whose peaks, although much lower than the main one, was observed in this type of individuals (see especially 2021 season in Fig. 4). These secondary peaks completely mismatched with the presence of pollinators, i.e., these late flowers did not find pollinators to visit them, so their reproductive success was practically zero.

This study shows that the main threat to this species is vertebrate predation, as it is for other *Scrophularia* species (e.g., *S. arguta*, Rodríguez-Riaño et al. 2022). Predation by vertebrates has been much more intense and damaging than that by invertebrates (caterpillars); the latter type of predation seems to be innate to many *Scrophularia* species (e.g., *Scrophularia frutescens* L., *S. arguta*, *Scrophularia canina* L., *Scrophularia lyrata* Willd., *Scrophularia nodosa* L., *Scrophularia xanthoglossa* Boiss., etc., Rodríguez-Riaño et al. 2022 and authors’ personal observations.). As mentioned, this greater intensity caused a flowering time-pollinator disruption in San Serván, an effect not observed in Cornalvo where predation by caterpillars did not disrupt this relationship; the lower level of predation by vertebrates in this population is probably explained by its location within a natural park, which ensures a higher level of protection. Long term, high density livestock activity (2021 season) could lead to the extinction of these natural populations or relegate them to even smaller areas with fewer individuals.

As with *S. arguta*, another species found in Extremadura that suffers serious predation by vertebrates, the need for conservation of *S. oxyrhyncha* is at a similar point, since the high density of domestic livestock, as well as that of wild vertebrates, endanger species or certain populations of these species; this does not generally arouse much interest in the general public because these species cannot be profitably exploited or because the situation of their population is not well known (due to a lack of research); this could lead to the loss of plant diversity, especially those species that are rare or have a small distribution area. These types of species should be considered a priority when it comes to conserving plant diversity, since their rarity or scarcity frequently coincides with very high livestock pressure that accelerates the entire degradation process of these populations/species and can drive them to extinction.

## Conclusions

There are at least two factors that potentially threaten the endemic Extremadura populations of *S. oxyrhyncha* endangering their survival: the first and most severe is cattle grazing density, which is responsible for a marked decrease in reproductive success that may lead to population collapse; second, and related to the previous factor, is the disruption of flowering time and pollinator activity, provoking pollen limitation and thus reducing plant reproductive success. We recommend that the regional/national authorities responsible for biodiversity take measures to conserve the populations of *S. oxyrhyncha*. These measures could include fencing, although this does not always have a positive effect on conservation (see Lorite et al. 2021). We consider that some population sectors could be temporarily fenced off and monitored to see whether cattle predation, which is the greatest threat, is reduced compared with unfenced sectors; we know this to be the case, because we fenced some plants to avoid predation, and the effect was positive. Second, both quartzitic and granitic populations of *S. oxyrhyncha* should be preserved ex situ in regional and national germplasm banks. Finally, a population dynamics study is in progress to monitor the evolution of these populations over time. Likewise, a population genetic study covering much more populations is underway to discern the possible causes that explain their morphological and longevity differences as well as their different level of susceptibility to predation. Since herbivory by cattle grazing could have a positive effect by reducing the level of geitonogamy, there needs to be a decrease in the cattle load in each population in order to achieve a certain trade-off between both factors.

## Electronic supplementary material

Below is the link to the electronic supplementary material.

Supplementary Material 1

## Data Availability

Data are available in the corresponding author upon request.
