# Supplementary material for: Knowing the reproductive biology and reproductive success of Scrophularia oxyrhyncha Coincy as a first step for its conservation
Source: Bot Stud. 2025 Jul 2;66:18. doi: 10.1186/s40529-025-00467-x (PMC12222582; doi:10.1186/s40529-025-00467-x)
Supplement: Supplementary file 1 — Supplementary Material 1 [file 40529_2025_467_MOESM1_ESM.docx]

**Knowing the reproductive biology and reproductive success of *Scrophularia oxyrhyncha* Coincy as a first step for its conservation**: Rodríguez-Riaño T, López E, López J, Pérez-Bote JL, Núñez B, Valtueña FJ, Ortega-Olivencia A

| **Table S1** Population and individual synchrony for the two *Scrophularia oxyrhyncha* populations using the Augspurger’s coefficient adapted to weeks | | | | |
| --- | --- | --- | --- | --- |
| **Population** | **Year** | **N** | **Synchrony** | |
|  |  |  | **Population** | **Individual** |
| San Serván | 2019 | 39 | 0.798 | 0.968-0.630 |
|  | 2020** | 40 | 0.881 | 0.987-0.749 |
|  | 2021 | 37 | 0.777 | 0.880-0.611 |
|  | 2022 | 35 | 0.759 | 0.871-0.657 |
| Cornalvo* | 2019 | 20 | 0.874 | 0.940-0.751 |
|  | 2021 | 41 | 0.847 | 1.000-0.777 |
|  | 2022 | 25 | 0.846 | 0.958-0.706 |
| Individual synchrony: *X_i_* = [1/(*n*-1)](1/*f_i_*)Σ*e_j≠i_*, where *j≠i* is the number of weeks in which individuals *j* and *i* coincide in flowering; *fi* is the number of weeks that individual *i* is in flower, and *n* is the number of individuals studied per population.  Population synchrony: *Z* = (1/*n*) Σ*X_i_*.  In both cases, unity represents maximum synchrony and zero the total absence of it.  *: Only individuals that flowered for at least 3 weeks were used.  **: Data are missing for 11 weeks (03/31/20 to 05/14/20) due to COVID-19 confinement. | | | | |

| **Table S2** Population and individual synchrony of flowering considering the time at which predation occurs for the San Serván population of *Scrophularia oxyrhyncha* using the Augspurger’s coefficient adapted to weeks | | | | |
| --- | --- | --- | --- | --- |
| **Year** | **Predation type** | **N** | **Synchrony** | |
|  |  |  | **Population** | **Individual** |
| 2019 | No | 5 | 0.784 | 1.000-0.625 |
|  | Late | 34 | 0.763 | 0.908-0.619 |
|  |  |  |  |  |
| 2021 | No | 17 | 0.829 | 0.945-0.663 |
|  | Yes | 20 | 0.774 | 0.932-0.668 |
|  | Late | 4 | 0.912 | 0.958-0.895 |
|  | Early | 12 | 0.722 | 0.883-0.612 |
|  | Double | 4 | 0.850 | 1.000-0.689 |
|  |  |  |  |  |
| 2022 | No | 21 | 0.807 | 0.894-0.729 |
|  | Yes | 14 | 0.692 | 0.800-0.615 |
|  | Late | 10 | 0.855 | 0.967-0.778 |
|  | Early | 4 | 0.569 | 0.833-0.467 |
| No, no predation observed; Yes, any predation type observed; early, predation before reaching peak flowering; late, predation after main peak flowering; double, early and late predation. | | | | |
